# The impact of the new definition of epilepsy on diagnosis, treatment, and short-term outcomes—A prospective study

**DOI:** 10.3389/fneur.2025.1564680

**Published:** 2025-03-24

**Authors:** Lena Habermehl, Louise Linka, Kristin Krause, Alena Fuchs, Jenny Weil, Mariana Gurschi, Felix Zahnert, Leona Möller, Katja Menzler, Susanne Knake

**Affiliations:** ^1^Epilepsy Center Hessen, Philipps-University Marburg, Marburg, Germany; ^2^Epilepsy Center, Neurological Institute, University Hospitals Cleveland Medical Center, Cleveland, OH, United States; ^3^Center for Neuroradiology, Philipps-University Marburg, Marburg, Germany; ^4^Center for Mind, Brain and Behavior, CMBB, Philipps-University Marburg, Marburg, Germany

**Keywords:** first seizure, ILAE, definition of epilepsy, seizure freedom, diagnostic criteria for epilepsy

## Abstract

**Background:**

In 2014, the ILAE introduced a new definition of epilepsy that allows some patients to be diagnosed earlier than under the previously used definition. According to the old classification, the diagnosis was made after a second unprovoked seizure. The risk of this was 36% after the first seizure. The aim of this study is to investigate the clinical impact of the new definition on diagnosis, treatment, and short-term outcome.

**Methods:**

From 2018 to 2021, adult patients admitted with a first epileptic seizure were prospectively included. Demographic and clinical data were collected at baseline, at 6 and 12 months follow-up (FU). Factors affecting seizure recurrence, especially age, use of anti-seizure medication (ASM), interictal epileptiform discharges (IED) in the EEG, and the presence of structural lesions on imaging were investigated.

**Results:**

Data from 235 patients were collected (41.7% female). Of these, 146 patients (62.1%) were diagnosed with epilepsy (PWE), following the new ILAE-criteria. Potential epileptogenic lesions on imaging were found in 49.3% of PWE. At the first FU (6.08 months ± 1.35), 143 patients (77.3%) were seizure-free, including 89 of the 146 patients diagnosed as PWE were seizure-free (70.6%). At the second FU (12.45 months ± 1.83), 129 patients (80.6%) were seizure-free. Seventy-seven of the PWE were seizure-free (72%). The use of ASM decreased (odds ratio = 0.46, *p* = 0.004) the recurrence rate significantly.

**Conclusion:**

Our results suggest that the new definition of epilepsy results in a higher frequency of epilepsy diagnosis and treatment. Short-term outcomes improved (1-year-recurrence rate of 19.4%).

## Key points

- Applying the new definition of epilepsy, 62.1% of the patients presenting with their first epileptic seizure were diagnosed with epilepsy.
- Among the newly diagnosed epilepsy patients, 78% achieved seizure freedom at 12-month follow-up.
- Treatment with anti-seizure medication was initiated in 66.3% of patients presenting with a first epileptic seizure.

## 1 Introduction

Epilepsy is a prevalent neurological disorder affecting ~0.7% of the general population (25), while the lifetime prevalence of an isolated epileptic seizure is much higher (8–10%) (26). Most population-based studies published so far are based on the old definition of epilepsy that was valid until 2014. They have shown a recurrence risk after a first epileptic seizure of ~36% after 1 year and 40–50% after 2 years (1–3). In 2014, the International League Against Epilepsy (ILAE) revised the definition of epilepsy (4). Epilepsy can now be diagnosed if at least two unprovoked (or reflex) seizures have occurred more than 24 h apart, if an unprovoked (or reflex) seizure occurs with a condition that increases the likelihood of further seizures within the next 10 years to more than 60%, or if an epilepsy syndrome is diagnosed (4). Pathological findings in EEG or MRI indicate a recurrence risk of over 60% over 10 years (4–8). Since 2014, this new definition is used worldwide (27), but its clinical impact remains unknown. Previous studies based on the former definition have suggested that antiseizure medication (ASM) therapy after an isolated epileptic seizure may reduce recurrence the risk of recurrence within the first 2 years, but may not improve long-term prognosis (9–11). However, these studies have primarily investigated the effects of older ASMs, and data on the long-term effects of newer ASMs remain limited. The present study aims to prospectively investigate the impact of the revised definition of epilepsy on diagnosis, treatment, and recurrence rates.

## 2 Methods

Patients who presented with a first epileptic seizure at Marburg University Hospital between February 2018 and January 2022 were prospectively included. All patients were at least 18 years old, and able to give informed consent were asked to participate in the study. The majority of patients were recruited during their inpatient stay directly after their first epileptic seizure. A few were recruited from the epilepsy outpatient clinic, with a delay of at least 1 week. Those whose diagnosis was changed to a non-epileptic diagnosis (migraine, syncope, etc.) after completion of the history and diagnostics were excluded.

Demographic data including age and gender, as well as clinical data such as comorbidities, semiology, EEG, CT, and 1.5-Tesla MRI results, discharge diagnosis (epilepsy, unprovoked first seizure, first acute symptomatic seizure), initiation of ASM, and the occurrence of seizure recurrence were collected at follow-up. The patients were scheduled for follow-up visits at 6 and 12 months. The study was approved by the local Institutional Review Board and followed the STROBE guidelines to minimize methodical bias.

### 2.1 Statistical analyses

Statistical analyses were performed using SPSS Statistics version 27 (IBM, 2020). Descriptive statistics were used to determine the relative frequencies of clinical characteristics in the sample. Descriptive statistics are presented as absolute numbers with percentages, means (M) with standard deviations (SD). To explore factors associated with seizure recurrence, a binary logistic regression was performed with the occurrence of a second seizure (yes/no) as the dependent variable. Independent variables included the use of ASM, age, interictal epileptiform discharges (IEDs) on EEG, and the presence of structural lesions on MRI or CT scans.

## 3 Results

In this study, we prospectively included 235 patients who presented with first epileptic seizures, of whom 41.7% were female, 58.3% were male. Applying the revised 2014 diagnostic criteria and definition of epilepsy, 62.1% (*n* = 146) met the criteria for epilepsy, while only 19.1% (*n* = 45) were diagnosed with epilepsy using the former criteria that were valid until 2014 (Table 1).

**Table 1:** Patient characteristics.

			***n***	**%**
**Age**, *M* ± SD	56.84 ± 21,61 years		235	
**Sex**	Male		137	58.3
	Female		98	41.7
**Death** within follow-up (FU) period			28	11.9
**Clinical diagnosis of epilepsy** after first seizure			146	62.1
Clinical diagnosis of epilepsy following old classification			45	19.1
**Epilepsy classification**				
Idiopathic generalized			12	
Focal			129	
Unclassified			5	
**EEG**			230	97.9
Epileptiform discharges			51	22.2
**Imaging**				
**MRI**			186	79.1
Epileptogenic lesion			59	31.7
Postischemic encephalomalacia			33	
Posthemorrhagic encephalomalacia			7	
Tumor			27	
Cortical dysplasia			3	
Hippocampal sclerosis			1	
**CCT**			49	20.9
Epileptogenic lesion			26	53.1
**EEG** **+** **MRI/CT**			230	97.9
Epileptiform discharges + epileptogenic lesion			22	9.6
**ASM**				
	**ASM**	**No ASM**		
PWE	135	11	146	
PWNE	21	68	89	
Monotherapy			147	
Combination therapy			9	
Levetiracetam			98	
Lacosamide			35	
Lamotrigine			15	
Brivaracetam			5	
Eslicarbamazepine			4	
Valproic acid			3	
Oxcarbazepine			1	
**Clinical presentation of first seizure**				
Acute symptomatic seizure			46	19.6
Status epilepticus			37	15.7
**Outcome**				
**6-months FU**, M ± SD	6.08 months ±1.35		185	
Lost to FU			50	
Seizure-free			143	77.3
PWE			126	
Seizure-free			89	70.6
PWNE			59	
Recurrent seizure			5	8.5
**12-months FU**, M ± SD	12.45 months ±1.83		160	
Lost to FU			75	
Seizure-free			129	80.6
PWE			107	
Seizure-free since last FU			77	72.0
Recurrent seizure since last FU			30	28.0
PWNE			53	
Recurrent seizure since last FU			0	0

Among patients directly diagnosed with epilepsy (PWE), 33.6% had interictal epileptiform discharges (IED) in EEG. Potential epileptogenic lesions on imaging (MRI or CT) were found in 49.3% of the PWE. EEG and imaging were performed in 142 PWE at the first visit, with four having no EEG. Of those, 20 PWE (13.6%) had pathological findings on both modalities (EEG and imaging). In addition, 45 patients showed no abnormal findings on both modalities, but later reported previously experienced aura symptoms or experienced conscious focal seizures and were therefore diagnosed with epilepsy (30.8%). The most common epileptogenic lesion was post-ischemic encephalomalacia (*N* = 33), followed by tumor lesions (*N* = 27) (such as brain tumors or metastases).

Of the 146 patients diagnosed with epilepsy (PWE), 135 (92.5%) were treated with ASM. Twenty one patients, who did not meet the diagnostic criteria for epilepsy (PWNE), treatment was initiated based on individual considerations such as patient request or increased risk of injury. Of these, 11 patients were treated after an acute symptomatic seizure, and seven patients were treated following status epilepticus as their first seizure manifestation. Levetiracetam was the most frequently chosen drug (*N* = 98), followed by lacosamide (*N* = 35) (Table 1).

### 3.1 Outcome

**Figure 1:**
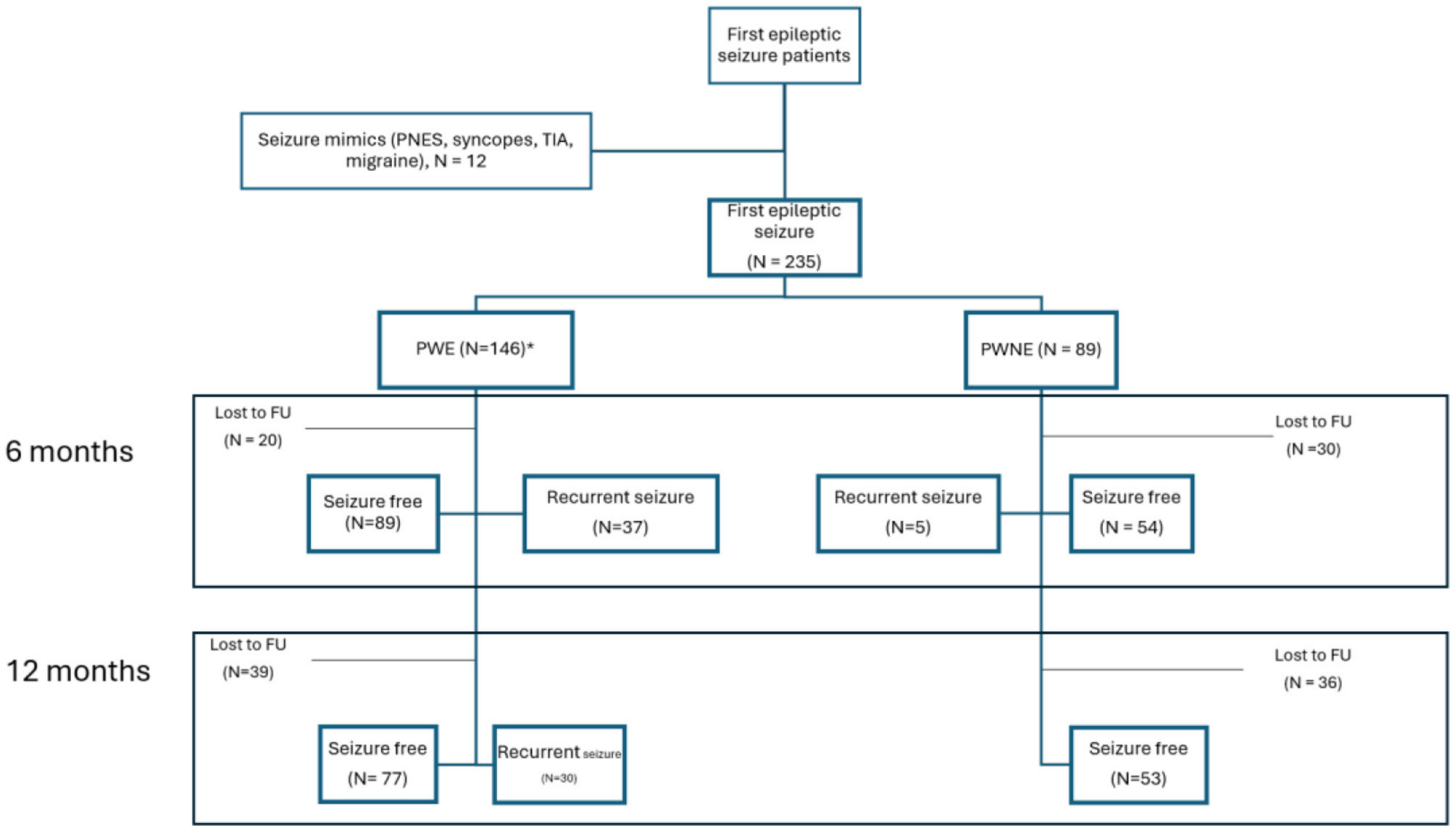
Outcome and lost-to-FU at first and second FU (*incl. 45 patients with recurrent undiagnosed seizures).

At the first follow-up visit (6.08 months ± 1.35), 50 patients (24 PWE, 26 PWNE) were lost to follow-up, and data from the remaining 185 patients (126 PWE, 59 PWNE) were analyzed and presented in Figure 1. Of these, 143 patients (77.3%) remained seizure free. Among the PWE, 89/126 (70.6%) were seizure-free, while 5/59 patients (8.5%) who did not meet the criteria for epilepsy after their first seizure had a second seizure by this time.

At the second follow-up (12.45 months ±1.83), data from 160 patients (106 PWE, 54 PWNE) were included in the analysis as 75 patients were lost to follow-up (35 PWE, 40 PWNE), as shown in Figure 1. Overall, 129/160 patients (80.6%) were seizure free. Among the PWE, 77/106 (72.6%) were seizure free. It is noteworthy that none of the patients who were not diagnosed with epilepsy after the first seizure suffered a second seizure between the 6 months and 1 year of follow-up.

In summary, in our cohort, the recurrence rate after the first seizure at 1 year follow-up was 19.4% at one-year follow-up for all patients, and 28% for patients with a diagnosis of epilepsy.

### 3.2 Risk factors for recurrent seizures

At the first follow-up visit, 182 patients (77.4%) were included in the analysis, and at the second follow-up visit, 156 patients (66.4%) met the inclusion criteria. There were no missing values for the selected predictors, and all assumptions for binary logistic regression were met. The predictors included age, use of ASM, presence of IED in EEG, and presence of structural lesions on MRI/CT scans. The full model yielded significant results at the first follow-up visit [χ^2^(3, *n* = 182) = 14.15, *p* = 0.007] and at the second follow-up visit [χ^2^(3, *n* = 156) = 24.66, *p* = 0.001], indicating that both models were able to discriminate between patients likely to experience a recurrent seizure and those who were not. At the first follow-up visit, the model explained between 7.5% (Cox and Snell R square) and 11.3% (Nagelkerke R square) of the variance in recurrence risk and correctly identified 76.9% of the cases. The logistic regression model, including age, ASM, IED in EEG, and structural lesions on MRI/CT scans as predictors, was able to discriminate between patients with and without recurrent seizures at both first and second follow-up visits (FU). The model explained 14.6% (Cox and Snell R square) to 23.7% (Nagelkerke R square) of the variance in recurrence risk and accurately identified 81.4% of cases at the second FU.

At first FU, the use of ASM was found to be a significant predictor (*p* = 0.001), with an odds ratio of 0.17 (95% CI.06–0.49), indicating that, when controlling for the other predictors, the odds of recurrence were five times lower with ASM treatment compared to those who did not receive ASM treatment.

At second FU, two predictors contributed significantly to the model: use of ASM (*p* = 0.004) and age at baseline (*p* = 0.034). The use of ASM was associated with a lower risk of recurrence, with an odds ratio of 0.046 (95% CI.06–0.37), highlighting its effectiveness in preventing recurrent seizures. Lower age was associated to yield a slightly increased risk of recurrence, with an odds ratio of 1.02 (95% CI 1.00–1.05). Neither the presence of IED on EEG, nor the presence of structural lesions on MRI/CT scans reached statistical significance at either the first or the second date of FU.

Of the 235 patients, *n* = 197 (83.8%) reported whether they had a recurrent seizure (*n* = 58 patients) and whether they were treated with ASM (*n* = 142, 72.1%) within the first year of FU. Fifty-eight patients had a second seizure within the period of FU, and 91.4% of them (*n* = 53) were treated with ASM. Only five of the untreated patients had a second seizure (9.9%) within the period of FU. Regardless the ASM, 89 (62.7%) of the treated patients had a second seizure within the period of FU (Table 2).

**Table 2:** ASM and seizure recurrence.

		**ASM**		
		**No**	**Yes**	**Total**
Recurrent seizure within FU-period	No	50	89	139
	Yes	5	53	58
	Total	55	142	197

## 4 Discussion

In this prospective study, we observed a markedly higher proportion of patients diagnosed with epilepsy and treated with ASMs after a single epileptic seizure, according to the current definition of epilepsy, compared to earlier population-based studies (1–4).

Surprisingly, our cohort had a favourable outcome, with 80.6% of patients being seizure-free after 1 year. To shed light on the possible reasons for this unexpected frequency of epilepsy diagnosis and the factors that contributed to this positive outcome, we aimed to identify relevant predictors in our dataset.

Fisher et al. (4) identified the presence of epileptogenic lesions on MRI or IED in the EEG as two factors leading to a higher risk for recurrent seizures after a first seizure. Similar to larger patient cohorts, such as those patients studied in the MESS trial, one fifth (22.2%) of all patients included in our study had IED in EEG (8, 10, 12, 13). In our cohort, only 33.6% of patients with epilepsy showed IED on EEG, which is lower than the 53% reported in the population-based Rochester study (12) and the 56% reported in the retrospective PRO-LONG study with a larger number of patients (6). The inclusion of children in both previous studies may explain this difference. This lower percentage of IED on EEG cannot explain the high frequency of epilepsy diagnoses observed in our patient cohort.

In our cohort, a relatively high proportion of patients showed abnormalities on imaging (36.2%), which contrasts with the lower rates of epileptogenic lesions reported in previous studies (ranging from 10% to 29%) (8, 10, 14). Furthermore, almost half (49.3%) of the patients with epilepsy in our study had potentially epileptogenic lesions on imaging, which is higher than the ~35% reported in a previous prospective study with a larger sample size (6). It is possible that our cohort included a higher proportion of patients with structural epilepsy, as our study only analysed adults, who are more likely to have such lesions compared to children.

### 4.1 Recurrence rate

According to previous literature, the risk of seizure recurrence after the first seizure has been reported to be 36% within the first year (1, 6, 8, 9). However, our cohort showed a lower recurrence rate of 19.4% for all patients and 28% for patients with epilepsy (PWE) after 1 year.

The lower risk of recurrence could be due to various factors, such as demographic differences between this study and earlier studies. Not including pediatric patients with epilepsies often caused by focal cortical dysplasia or children with perinatal neurological deficits, who are usually refractory to ASM, may explain the low risk of recurrence in this study (1, 15).

However, our recurrence rate seems to be in line with previous studies, which published a recurrence rate of 13% at 1 year in patients treated with ASM (16).

Several risk factors for seizure recurrence have been identified in previous studies. Pathological findings in the EEG have consistently been associated with an increased risk of subsequent seizures in multiple studies, although the specific definition of EEG abnormalities varies between authors. Some studies have focused solely on IED, while others have also reported a slowing of the EEG. In 2015, Krumholz et al. conducted a systematic review and reported that the presence of IED in the EEG was associated with a 2.16-fold increase in the rate of seizure recurrence between 1 and 5 years compared to patients without such abnormalities (1, 5–8). The presence of potential epileptogenic lesions in the MRI and a history of brain injury have also been shown to be significant risk factors for recurrent seizures. While Beretta et al. did not find every individual risk factor to be predictive alone, they described that the combination of abnormal EEG, imaging findings, and deficits in the neurological examination was associated with a higher risk of recurrent seizures (6, 8, 17). Additional risk factors for seizure recurrence have been identified, including the presence of focal or nocturnal seizures, a family history of epilepsy, and neurological deficits (18). Surprisingly, in our cohort, the presence of IED in the EEG or epileptogenic lesions on MRI did not significantly affect short-term outcomes, which is consistent with a few rare studies that failed to demonstrate these factors as reliable predictors of seizure recurrence (5, 19). This may be due to the use of ASM treatment, which may delay the onset of a second seizure, making the short follow-up period of this study insufficient to detect recurrent seizure (10).

Another plausible explanation for the low recurrence rate could be that the structural imaging findings are mainly ischaemic strokes (33/59 lesions), which have a relatively low epileptogenicity (20, 21). Notably, only 8.6% of patients, who experienced a second seizure during the follow-up period, did not receive ASM. It is noteworthy that all PWNE in the cohort experienced the second seizure within 6 month of the first. This finding supports the actual recommendation not to drive within 6 months after the occurrence of a first seizure in most European countries (22, 23).

Another reason for the favourable outcome of our cohort could be the early treatment with ASM after the first seizure.

### 4.2 Treatment

In the aforementioned systematic review, which analysed data from twelve studies (3,212 patients) published between 1982 and 2006 with a first unprovoked seizure were analysed, 43% of the patients were treated with ASM (9). In our cohort, 66.4% of the patients were immediately treated with ASM. The performed logistic regression indicates a decreased risk for seizure recurrence due to the use of ASM.

The efficacy of ASM in reducing seizure recurrence is well-established. However, the optimal time to start ASM treatment remains a subject of considerable debate. Several studies have reported a reduction in seizure recurrence within the first 2 years of treatment when ASM is administered immediately after the first seizure, ranging from 48% to 36% (10), 39% to 32% (5), and 40% to 32% (6, 16), compared with delayed administration after the second seizure. A systematic review of 12 studies published between 1982 and 2006 found that the frequency of ASM treatment after the first seizure was mostly < 50% (ranging from 13% to 69%) (9). All of these studies were conducted at a time when the old definition of epilepsy recommended treatment only after the occurrence of two unprovoked seizures.

Two controlled prospective studies, MESS and FIRST, compared immediate vs. delayed initiation of ASM, primarily using carbamazepine, valproic acid, phenytoin, and phenobarbital, and showed improved short-term outcomes (10, 16). In our cohort, 156 out of 235 patients (66.4%) received ASM treatment immediately after their first seizure, primarily using levetiracetam, lacosamide, and lamotrigin. As a result, a relatively low recurrence rate (19.4%) supports the efficacy of ASM in achieving favourable short-term outcomes.

Previous studies have shown differences in the long-term outcome of ASM treatment. The 2-year remission rate varied between 86% after 10 years (11) and a 2-year remission rate of 96% after eight years (10). However, the results of the two prospective, controlled studies (MESS and FIRST) did not support the notion that early initiation of ASM could improve the long-term outcome (9). The effect of newer ASMs on the long-term outcome needs to be investigated in future studies.

## 5 Limitations

The main limitation of our study is certainly its descriptive nature and the inherent lack of a control group. Thus, on the one hand, it remains unclear whether the high diagnosis rate of epilepsy is appropriate or whether abnormalities in imaging that justify the diagnosis are overestimated. The lack of a control group makes it is also impossible to clarify whether the low recurrence rate is actually due to the early drug therapy.

It therefore ultimately remains unclear whether the high diagnosis rate associated with a good outcome corresponds to a good response to ASM, or whether some of the patients were overdiagnosed and would have remained seizure-free with or without ASM.

The lack of follow-up inherent in a prospective study is also a limitation that needs to be considered. It is possible that some of the patients no longer appeared due to seizure freedom, but this is not necessarily the reason (24).

## 6 Conclusion

Our results suggest that the new definition of epilepsy results in a high frequency of epilepsy diagnosis and ASM treatment. Nevertheless, early detection and administration of ASM appears to result in favourable outcomes for people with epilepsy within the first year. The reasons for this remain unclear and could be due to the specific characteristics of the cohort (e.g., the higher mean age), or even a possible overdiagnosis of PWE. Case-control studies with a large number of cases and longer follow-up could address this.

Fisher et al. emphasized that the presence of imaging findings and IED on EEG does not necessarily result in a seizure recurrence risk of over 60%. In addition to diagnostic findings, clinical factors such as family history, nocturnal seizures, previous brain injury, and the presence of focal seizures should be considered when assessing the risk of seizure recurrence or seizure-related harm. Immediate initiation of ASMs is recommended only when there is a high risk of recurrence or risk of seizure-related injury or death. Knowing, that earlier diagnosis and treatment only impacts short-term outcomes and not long-term outcomes, physicians should discuss the potential social or economic consequences of a diagnosis, and potential side effects of treatment and counsel their patients on an individual level before starting an ASM (8, 9).

## Data Availability

The raw data supporting the conclusions of this article will be made available by the authors, without undue reservation.
